# Design and Implementation of a VoIP Broadcasting Service over Embedded Systems in a Heterogeneous Network Environment

**DOI:** 10.1155/2014/917060

**Published:** 2014-09-15

**Authors:** Jenq-Shiou Leu, Wei-Hsiang Lin, Wen-Bin Hsieh, Chien-Chih Lo

**Affiliations:** Department of Electronic and Computer Engineering, National Taiwan University of Science and Technology, Taipei 106, Taiwan

## Abstract

As the digitization is integrated into daily life, media including video and audio are heavily transferred over the Internet nowadays. Voice-over-Internet Protocol (VoIP), the most popular and mature technology, becomes the focus attracting many researches and investments. However, most of the existing studies focused on a one-to-one communication model in a homogeneous network, instead of one-to-many broadcasting model among diverse embedded devices in a heterogeneous network. In this paper, we present the implementation of a VoIP broadcasting service on the open source—Linphone—in a heterogeneous network environment, including WiFi, 3G, and LAN networks. The proposed system featuring VoIP broadcasting over heterogeneous networks can be integrated with heterogeneous agile devices, such as embedded devices or mobile phones. VoIP broadcasting over heterogeneous networks can be integrated into modern smartphones or other embedded devices; thus when users run in a traditional AM/FM signal unreachable area, they still can receive the broadcast voice through the IP network. Also, comprehensive evaluations are conducted to verify the effectiveness of the proposed implementation.

## 1. Introduction

In the recent decade, the function of data network transits from transmitting texts and images to transmitting real-time multimedia, like voice or video. The IP Multimedia Subsystem (IMS), introduced by the 3rd Generation Partnership Project (3GPP), was proposed to deliver the multimedia content over a fixed-mobile convergence (FMC) network [1] as a carrier-grade solution. Although nowadays multimedia services are pervasive [2], more and more people expect a low-cost, maybe even free technology to enable the real-time communication. VoIP is the most important application among many low-cost and real-time communication ones. However, the VoIP technique still encounters many challenges such as packet collision and loss due to common circuits, material delay [3]. The challenges may result in a poor voice quality. Furthermore, different software and hardware conditions at different end-points may also influence voice quality. In general, equipping the VoIP devices with a broadband network access can make a better voice quality achievable. Perceptual Evaluation of Speech Quality (PESQ) is a common index to show the voice quality [4], which compares the original input signal with the degraded output that is the result of passing through a communication system. The output of PESQ is to quantify the difference between the ideal input and the degraded output. It also represents the result in a subjective listening test.

Besides, some researches focus on the capacity of voice data over some specific wireless network, such as IEEE 802.11 [5, 6]. However, they paid less attention to the fact that users may access the Internet through heterogeneous networks nowadays. In [7] the embedded system architecture (ESA) supporting cellular and WLAN connections was used to deliver a high-performance mobile VoIP service. However, if we want to make more people who are far away from each other and accessing different networks hear the same voice in real time, a broadcasting function is required for converting analog voice signals into digital signals and then conveying digital signals to all listeners.

To the best of our knowledge, little effort was made to practically build a VoIP broadcasting service without the support of a carrier-grade solution. Besides, the broadcasting service is still an interesting application for the group communication. The traditional amateur radio application is restricted by the hardware and the spectrum. However, VoIP is run over the IP network and the IP network is ubiquitous in the fixed or mobile network nowadays. Our proposed concept can even be spread as an APP on smart phones which are widely penetrated in the modern communication world. This service can be regarded as one of APPs on the smartphones, instead of the conventional amateur radio application on a dedicated ham radio. Currently there exist many VoIP open source projects. In this paper, we show how to implement the VoIP broadcasting service over Linphone [8], one of VoIP open source projects, on the embedded systems and find an efficient way to deliver the voice packets to multiple users in heterogeneous networks.

The remainder of the paper is organized as follows. In Section 2, some related works are discussed and our main contribution is described. We introduce a VoIP open source—Linphone—used in our implementation and experiments in Section 3. In Section 4, the system architecture of our implementation is presented. The performance evaluation based on our implementation is illustrated in Section 5 and a brief conclusion is drawn in Section 6.

## 2. Related Works

In recent years, most of traditional communication media including telephone, music, film, and television are digitized to be transmitted over the Internet. Voice-over-Internet Protocol (VoIP) [9] is a family of technologies, communication protocols, and transmission techniques for delivering voice and multimedia over Internet protocol networks. As Skype has been released, many people are aware of the convenience of voice and data over the network. Since the mid-1990s, telephone equipment manufacturers have added IP capabilities to their existing PBX telephony switches. The VoIP phone provides an alternative solution to the traditional telephone. As the VoIP technology gets mature, how to get a better quality of services (QoS) of VoIP was studied. VoIP over wired networks has many breakthroughs. Walravens and Gaidioz [10] presented a technique called receive descriptor recycling (RDR) that can reduce small-packet loss by 40%. Also the most popular applications such as Skype therefore become the research focus [11, 12].

In 2001, in order to determine the influence of delay jitter and packet loss on the perceived quality, Duysburgh et al. [13] used an objective speech quality assessment system, DSLA, to predict the mean opinion score (MOS) for voice connections. On a connection without dejitter buffer, a devastating influence of jitter is shown whereas the influence on a connection with dejitter buffer is found to be similar to the effect of packet loss in the network. To observe the influence of packet loss, delay, and delay jitter as well as the effect of sampling, digitization, coding/decoding, and packetization, many experiments were carried out, such as [14–17]. Next, Oklander and Sidi [18] modeled a jitter buffer operation by M/G/1/K-ex queue and carried out the analysis of its performance. To increase the perceived quality of the voice, they used voice quality evaluation methodologies—MOS and E-model with the established relations between delay jitter and packet loss—to choose the controlling parameters for the jitter buffer setting optimally. Later, Paulsen et al. [19] investigated how the jitter buffer affects the QoS. Paulsen et al. carried out two implementations that consider a passive FIFO buffer and an active PJSIP buffer. They presented the results that demonstrated considerable improvements in VoIP QoS by doubling the size of jitter buffer. The study shows the dependency between QoS values, end-to-end delay, and the jitter buffer size.

Jang et al. improved the voice quality in a WLAN/cellular dual-mode mobile phone by proposing new embedded system architecture (ESA) [7]. Based on a dual-core scheme, main functional blocks of the proposed architecture are composed of VoIP remote procedure call (VRPC), an audio bridging scheme, and a server-assisted call management (SACM) algorithm. Jung and Ibanez [20] proposed an adaptive piggyback packet coding that can provide the VoIP user with an acceptable quality over the wide range of packet loss conditions.

Most of the above studies were done in a homogeneous network and focused on one-to-one communication. Therefore, we plan to present a broadcasting service over heterogeneous networks and evaluate the influence of broadcasting order and process scheduling on the QoS. Moreover, we place the emphasis on voice call since voice call is still the most common communication mode without invading privacy. Besides, the signal quality for the AM/FM broadcasting system is vulnerable, so that the voice broadcasting service quality in the traditional analog system is not stable. The IP-based digital voice can keep a good quality as long as the IP connectivity is fine. Even in the tunnel, network operators may use the radio-over-fiber, mesh network, and cellular network repeater technologies to extend the IP service coverage for keeping the communications service out of interruption. VoIP broadcasting over heterogeneous networks can be integrated into modern smartphones or other embedded devices. Therefore, users can receive the broadcast voice through the IP network almost anywhere.

In [21], multimedia push-to-talk service deployed indoors can monitor the inner environment and make communication among users in the area; however, the service is limited to a local area. Next, the authors in [22] explained that a push-to-talk issuer can use lower cost to extend the PTT service over the Internet by deploying VoIP broadcasting servers but did not evaluate the QoS based on a real voice quality. In [6], authors also discussed when using G.729 codec in VoIP, how many calls can be held at the same time over the IEEE 802.11b networks. However, they only considered the receivers are in the same distance. In this paper, we implement a VoIP broadcasting service by an open source project VoIP software—Linphone—on embedded systems and carry out and evaluate a VoIP broadcasting service in not only homogenous but also heterogeneous networks. Furthermore, we discussed the real received voice quality, delay, and how to optimize the system efficiency in the system evaluation experiment.

## 3. Introduction to Linphone

Linphone [8] is an open source project; its software implementation can be used to communicate freely with people over the Internet, with voice, video, and text instant messaging. Linphone uses the SIP protocol, an open standard for Internet telephony, for communication and is licensed under the GNU General Public License. And it is available for desktop computers running Linux, Windows, and MacOSX or smartphones running Android and iOS.  Table 1 illustrates the comparison of different VoIP software. Since Linphone can adopt different codec mechanisms and support various operating systems, we utilize Linphone over embedded systems to implement our VoIP broadcasting service.

There are two major parts in Linphone: one is the user interface and the other is the core engine. GTK+/glade is used for GUI and a console-mode application can also be executed on Linux. The core engine is Liblinphone which is the library implementing all the functionalities of Linphone. Liblinphone is a powerful SIP VoIP video SDK that can be used to add audio or video call capabilities to an application and provides a high level API to initiate, receive, or terminate calls. Multimedia communication is usually made of rich media (such as voice or video) and signaling (such as routing calls and accepting a call). Linphone aims at joining these two things up to implement voice/video calls in applications easily. Liblinphone depends on three software components—(1) Mediastreamer2, (2) oRTP, and (3) eXosip2.

1. Mediastreamer2: a library which is responsible for receiving and sending multimedia streams in Linphone, including voice/video capturing, encoding and decoding, and rendering. In brief, it is a powerful and light-weighted streaming engine specialized in voice/video telephony applications.
2. oRTP: a real-time transport protocol library which includes APIs to parse incoming RTCP packets. It supports multiple profiles and the AV (audio/video) profile is the default one. It also can support the SRTP (secure RTP) feature.
3. eXosip: a library which hides the complexity of using the SIP protocol for building multimedia sessions.

**Figure 1:**
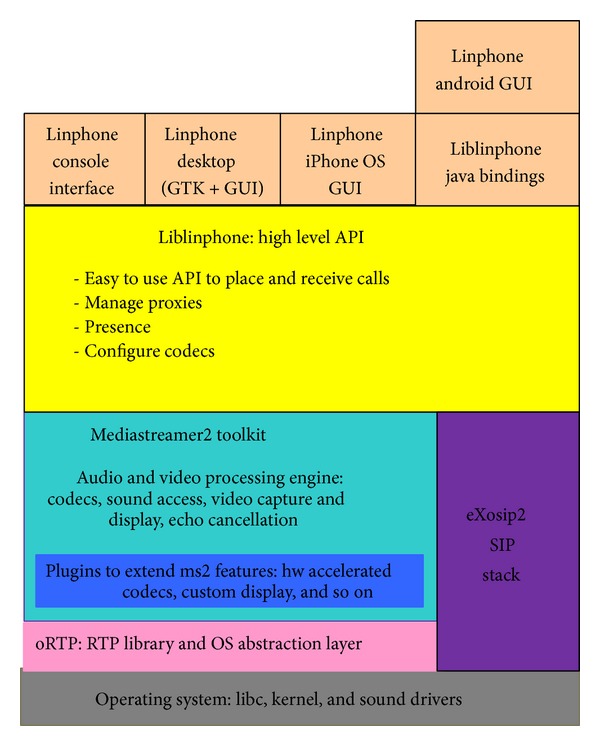
Software architecture of Liblinphone and all its dependencies [8].

The architecture of Linphone is shown in Figure 1. Linphone separates user interfaces from the core engine, allowing creating various kinds of user interfaces on top of the low tier functionalities.

## 4. Architecture of the Proposed VoIP Broadcasting Service over Heterogeneous Networks

**Figure 2:**
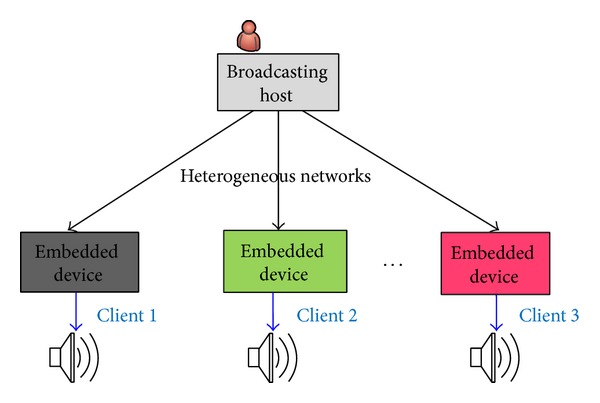
Conceptual architecture.

The main contribution of this paper is to implement a VoIP broadcasting system and use the VoIP technology to broadcast voice to diverse clients over heterogeneous networks. The conceptual architecture is shown in Figure 2.

**Figure 3:**
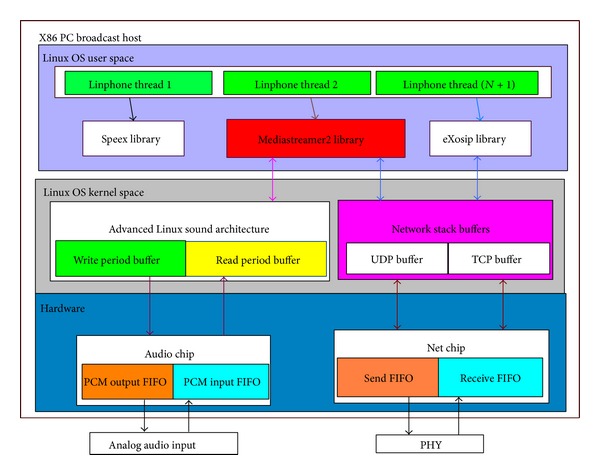
X86 PC broadcasting host.

**Figure 4:**
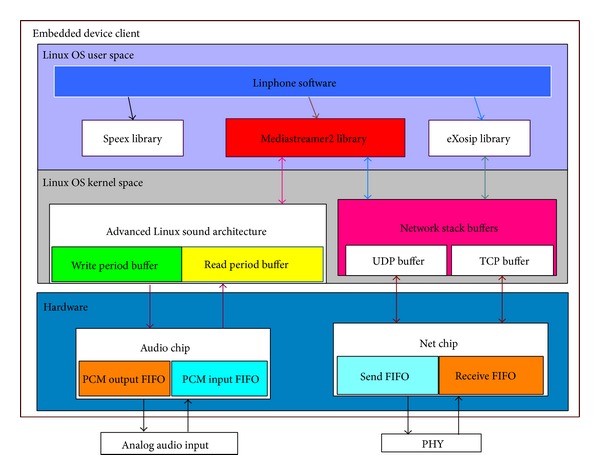
ARM-based embedded client.

The analog audio is inputted into the broadcasting host and is broadcasted to embedded device clients over heterogeneous networks, such as WiFi, LAN, and 3G networks. The detailed system architectures of the broadcasting host and the embedded device are illustrated in Figures 3 and 4.

In the implementation environment, the specification of the broadcasting host we use is a X86 PC with an Intel CPU T2060 1.60 GHz, 1 GB DDR RAM running Ubuntu 11.04 and the one of the embedded client is a S3C6410A board with a CPU, ARM1176JZF-S 533 MHz, and a 1 GB DDR RAM running Android 2.3.2 (based on Linux kernel 2.6.38). These specifications are subject to change based on the executive plan.

In the architecture of the broadcasting host, there exist many Linphone threads, in accordance with the numbers of receivers, running in the user space. Each thread shares the same memory, libraries, and OS resources. In the OS kernel space, ALSA (Advanced Linux Sound Architecture) provides Linux audio and MIDI (musical instrument digital interface) functions. Network stack buffers are used to store and send UDP/TCP packets. If the buffer is large, the operating system accumulates more packets and then passes the packets to the chip at a lower layer. In this way, the CPU is less busy, but the packet arrival may not be real-time. In contrast, the smaller the buffer is, the more real-time the packet arrival is. At the hardware layer, an audio chip is used to convert analog audio signal that sends to/receives from users and a net chip is used to send/receive digital audio packets. That is, the voice is extracted by an audio chip and stored in the buffer of the audio chip. Then the audio data is compressed by Linphone and divided into small packets. These packets then are moved to the buffer of the net chip and sent to clients through the network. On the client side, the architecture is similar to that of the host side. The difference is that only one thread runs on the client side, which corresponds to one of many threads on the broadcasting host. The audio data is extracted from the buffer of the net chip and is decompressed by Linphone first and then ALSA sends the data to the audio buffer to play the voice.

**Figure 5:**
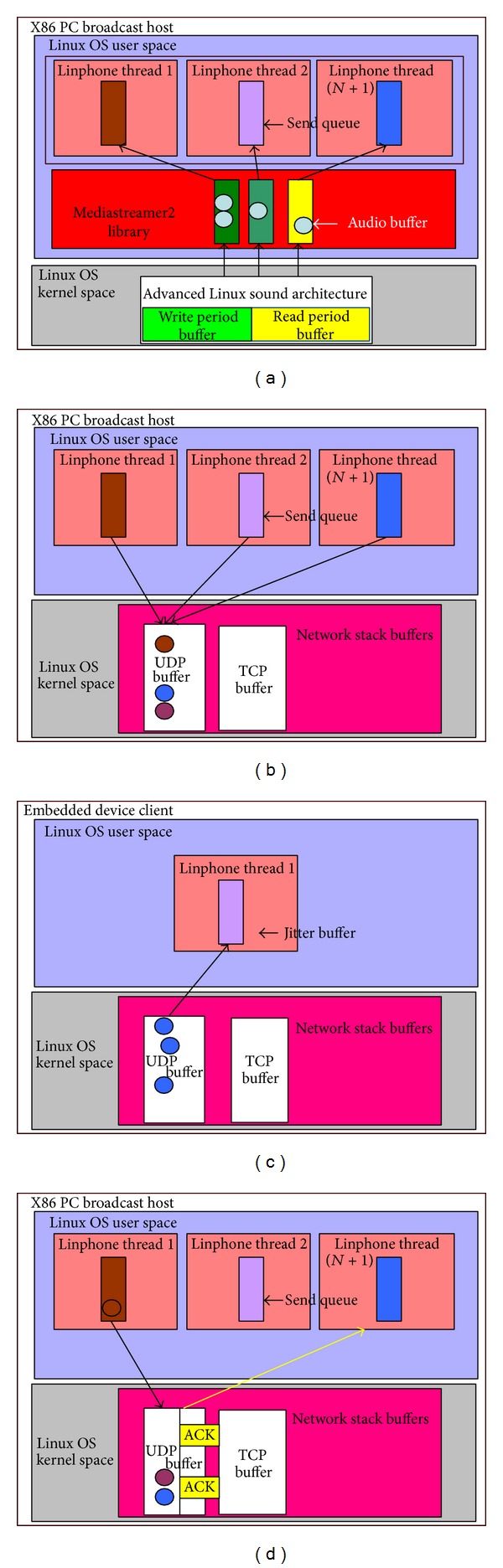
Audio broadcasting procedure.

Our proposed system architecture is run over heterogeneous networks. When delivering the audio content from the broadcasting host to the clients, each Linphone thread has its own audio buffer. The analog audio input would be converted into digital audio packets by using some codec like G.711, which is a common codec at the host side. When an audio buffer is full, it will send packets to the corresponding thread's sending queue which is in Linux OS user space. Each thread corresponds to a different embedded device. Then packets are sent to the UDP buffer in Linux OS kernel space. Next, each packet at the host side is sent to the jitter buffer on each embedded device at the client side. After receiving the audio packet, each embedded device sends an ACK response to the host and then the host would continue to send the following audio packets to the embedded devices when receiving the ACK. The process flow is sequentially illustrated in Figures 5(a), 5(b), 5(c), and 5(d).

Since many clients may currently receive the broadcast voice, the host side sends the voice packet to thread in a round-robin manner. If the host wants to transmit audio data to different network nodes that have a variety of transmission rates in a heterogeneous network environment, it is better to transmit voice packets to the better network node first and then to the inferior. The proposed shortest transmission time node first can be presented as in Algorithm 1.

## 5. Performance Evaluation

**Figure 6:**
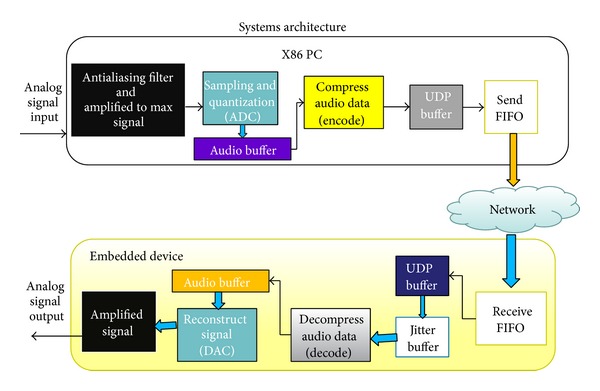
The physical architecture of signal conversion.

Linphone implements part of RTCP (real-time control protocol) so that the receiver would send an ACK back to the sender when it receives the voice packets. Hence, the Linphone-based broadcasting host can know (1) how many packets have been sent, (2) how long the transmission delay of voice packet is, and (3) which delayed packet should be discarded.  Figure 6 show the simple procedure in our experiment that converts analog audio input into digital audio signal, encoding the signal at the X86 PC-based sender, decoding the signal, and finally turns to the original audio output at the embedded device-based receiver.

**Figure 7:**
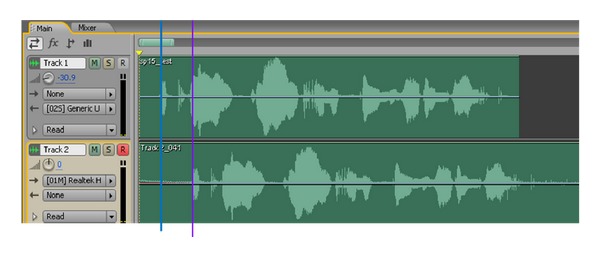
Adobe Audition player.

Measuring the delay of packet transmission through the timestamps of packets sent or received over the Internet may not be precise due to the influence of clock skew (clock offset of devices) [23]. Therefore, we use the Adobe Audition player which can record and play audio simultaneously (shown in Figure 7). Through playing the audio on the broadcast host and recording the audio at the receiver side, the transmission delay can be measured by the Adobe Audition player itself.

**Figure 8:**
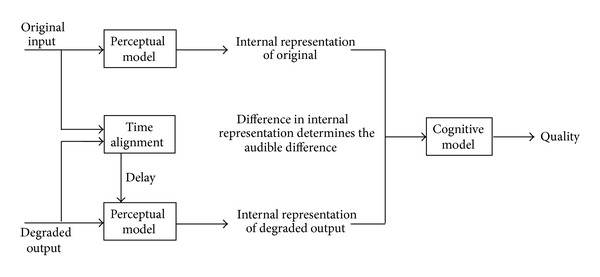
PESQ measure process.

Next, we use PESQ measure program to calculate the difference between the received audio and the original audio to obtain the PESQ value (shown in Figure 8).

### 5.1. Received Voice Quality on Different Kinds of Embedded Devices

The first experiment illustrates the evaluated performances of sending VoIP packets from a broadcasting host (an x86 PC running on Linux) to two different kinds of embedded devices—to be a host—and two embedded devices (one running on the Linux kernel and the other running on the Android platform) in the WiFi environment. In the experiment, the audio buffer size is set to 20 ms that is equal to the frame size in G.711. We use the transmission delay and PESQ as the measurement indices to assess the received qualities on these two receivers. In 1,000 experiments, we got the evaluation results shown in the Tables 2 and 3 by taking the average delay time and PESQ of all experiments. The result in Table 2 shows a Linux-based embedded device which receives a better service quality than an Android-based one.

We can see that the performance of the pure Linux kernel device is better than that of Android-based one since the Android-based embedded devices have one additional application layer that is used to transfer the audio to the kernel. According to the rating of PESQ, the satisfied voice quality should have PESQ above 3.5. Therefore, both still have an acceptable quality of voice.

### 5.2. VoIP Broadcasting over Heterogeneous Networks

**Figure 9:**
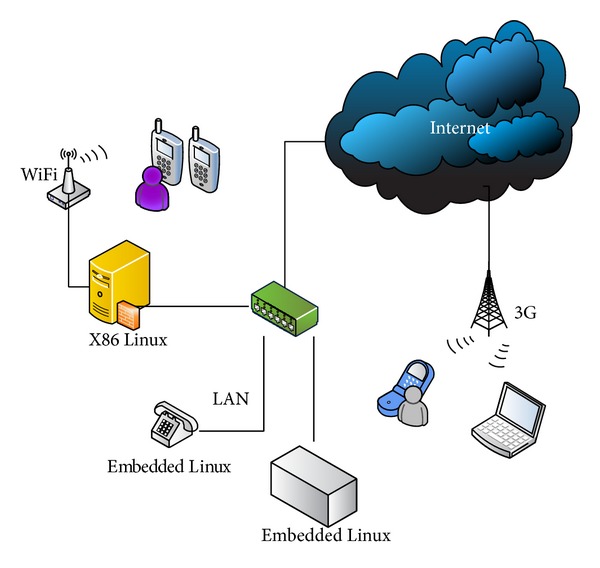
The deployment over heterogeneous networks.

In our second experiment, we conduct VoIP broadcasting over heterogeneous networks, including LAN, WiFi, and 3G. Its deployment is shown in Figure 9.

The embedded devices are deployed in different networks; the x86 based broadcasting host can communicate with these devices through LAN, WiFi, or 3G network. For perception of human, the real-time voice must be delivered in less than 50 ms to make the communication smooth. Therefore, the broadcast packets must reach each device in the restricted time.

In Table 3, we present the access network and status for each device. Different circumstances would affect the service quality. For example, the network quality in 3G is worst so that the device in the 3G environment may take more than 100 ms average delay to receive the broadcast packets. Therefore, we try to schedule threads' priorities to broadcast voice packets in different sequences on the x86-based host for finding a better service quality for all devices.

**Figure 10:**
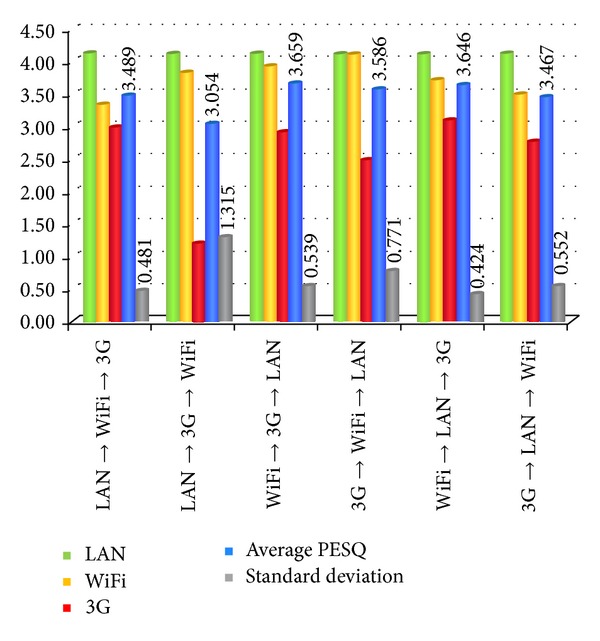
The result of different broadcasting sequences.

Recalling that our broadcasting architecture needs to wait for finishing sending the same packet to each embedded device, the following packet may be sent then. As the result in Figure 10 shows, we can find when the devices in a better transmission condition are sent with a higher priority, all received voices can reach an acceptable quality with just little variances among all devices due to a lower PESQ standard deviation.

**Figure 11:**
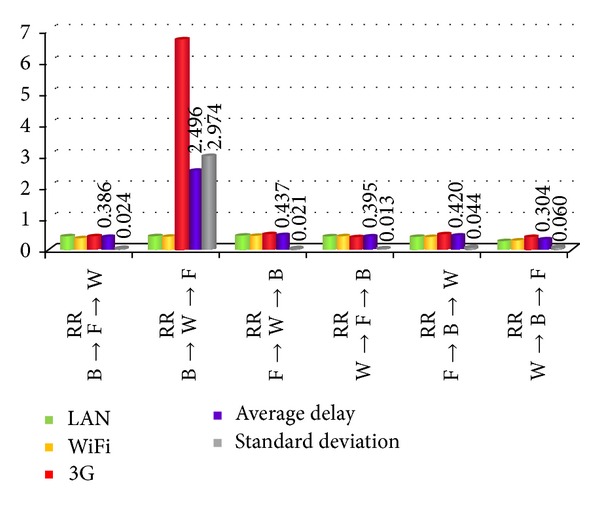
The result of different packet broadcasting sequences.

As the result in Figure 11 shows, B (best) means LAN, F (fair) means WiFi, and W (worst) means 3G. We can find that if the worst transmission condition is sent with a higher priority, we can get a better result in average delay among all devices.

### 5.3. Simulation Evaluation of Real-Time Multiple Receiver Nodes

**Figure 12:**
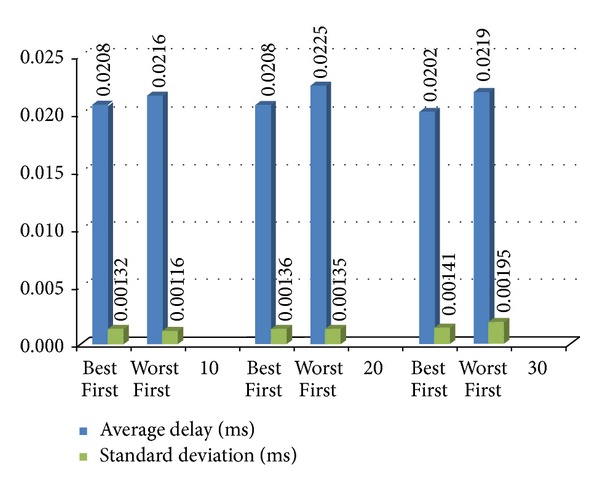
The result of multinode broadcasting.

To verify the service under a larger volume of receiver node, we use VMware virtual machines running on Linux to simulate multiple receiver nodes attached to virtual network interface cards with virtual IP addresses. In order to simulate different network environments, we purposely delay the transmission rates on each network interface. Then we use the “ping” command to produce 160-byte packets which are the same as audio packets following the G.711 codec rule and send these packets to test the transmission rates of these receiver nodes. In our experiment, we conduct 10 to 30 different network environments with different transmission rates. By setting round-robin scheduling on Linux, we change the priority of sending packets to these testing nodes. As the result shown in Figure 12, we can find that the average delay with the assistance of the proposed STTNF scheme (Best First) is lowest compared to the one with an opposite sequence (Worst First).

## 6. Conclusion

VoIP has become a popular technology in modern communication. Many free softphones such as Skype, Yahoo! Messenger, and Google Talk are available now. These softphones provide high-quality voice services. However, most of the existing applications do not consider what the network environment is. Not much effort is paid to deep research in VoIP over heterogeneous networks, not to mention broadcasting over them. Broadcasting VoIP to multiple receivers, which may be built on the diverse embedded system and smartphones, can be a complementary service for the amateur radio application, especially when people enter a radio unreadable area. In this paper, we use free software—Linphone—to practically establish the broadcasting architecture and evaluate the influence of audio buffer, jitter buffer in such a service. Then we propose a STTNF scheme to broadcast voice packets to multiple embedded system-based receivers in heterogeneous networks' environments. Meanwhile, we conduct comprehensive experiments to verify the broadcasting service quality over heterogeneous networks, including LAN, 3G, and WiFi networks.

## Figures and Tables

**Algorithm 1:**
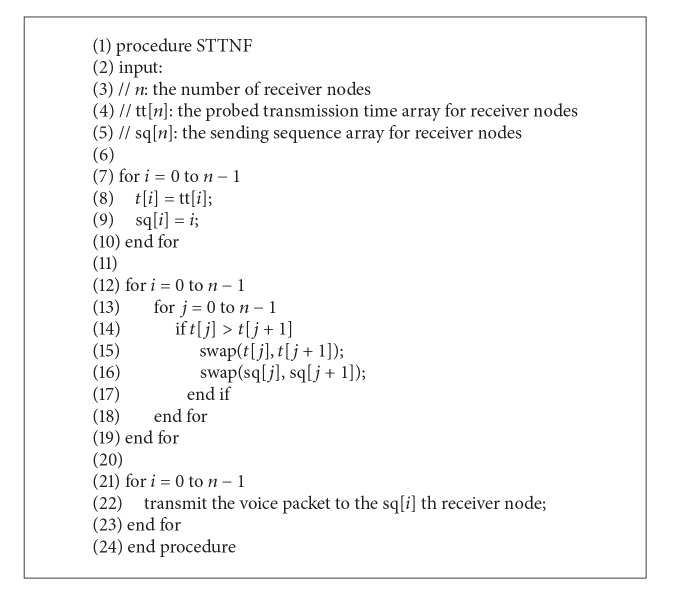
Shortest transmission time node first (STTNF) pseudocode.

**Table 1:** The comparison of current VoIP software.

2012/06 for VoIP voice support	Skype	Yahoo and MSN Messenger	Google Talk	Line	Linphone
Open source	No	No	No	No	Yes
Select and change audio codec	No	No	No	No	Yes
Free to use	Yes	Yes	Yes	Yes	Yes
OS Support					
Android	Yes	Yes	Yes	Yes	Yes
BlackBerry	Yes	No	Yes	No	Yes
Linux	Yes	No	Yes	No	Yes
Windows	Yes	Yes	Yes	Yes	Yes
MacOSX	Yes	Yes	Yes	Yes	Yes
iPhone	Yes	Yes	Yes	Yes	Yes

**Table 2:** The delay and PESQ between the host and the receivers.

	Linux-based embedded device	Android-based embedded device
Delay (ms)	340	440
PESQ	4.04	3.87

**Table 3:** The status description of each device.

Access network	LAN	WiFi	3G
Average delay	0.303 ms	1.947 ms	113.429 ms
Delay mean deviation	0.024 ms	0.612 ms	16.801 ms
OS	Embedded Linux	Embedded Linux	Win7
CPU	ARM11	ARM11	Atom
